# Past, present and future distributions of Oriental beech (*Fagus orientalis*) under climate change projections

**DOI:** 10.1371/journal.pone.0242280

**Published:** 2020-11-17

**Authors:** Dilsad Dagtekin, Evrim A. Şahan, Thomas Denk, Nesibe Köse, H. Nüzhet Dalfes

**Affiliations:** 1 Eurasia Institute of Earth Sciences, Istanbul Technical University, Istanbul, Turkey; 2 Department of Paleobiology, Swedish Museum of Natural History, Stockholm, Sweden; 3 Department of Forest Botany, Faculty of Forestry, Istanbul University-Cerrahpasa, Istanbul, Turkey; Technical University in Zvolen, SLOVAKIA

## Abstract

Species distribution models can help predicting range shifts under climate change. The aim of this study is to investigate the late Quaternary distribution of Oriental beech (*Fagus orientalis*) and to project future distribution ranges under different climate change scenarios using a combined palaeobotanical, phylogeographic, and modelling approach. Five species distribution modelling algorithms under the R-package `biomod2`were applied to occurrence data of *Fagus orientalis* to predict distributions under present, past (Last Glacial Maximum, 21 ka, Mid-Holocene, 6 ka), and future climatic conditions with different scenarios obtained from MIROC-ESM and CCSM4 global climate models. Distribution models were compared to palaeobotanical and phylogeographic evidence. Pollen data indicate northern Turkey and the western Caucasus as refugia for Oriental beech during the Last Glacial Maximum. Although pollen records are missing, molecular data point to Last Glacial Maximum refugia in northern Iran. For the mid-Holocene, pollen data support the presence of beech in the study region. Species distribution models predicted present and Last Glacial Maximum distribution of *Fagus orientalis* moderately well yet underestimated mid-Holocene ranges. Future projections under various climate scenarios indicate northern Iran and the Caucasus region as major refugia for Oriental beech. Combining palaeobotanical, phylogeographic and modelling approaches is useful when making projections about distributions of plants. Palaeobotanical and molecular evidence reject some of the model projections. Nevertheless, the projected range reduction in the Caucasus region and northern Iran highlights their importance as long-term refugia, possibly related to higher humidity, stronger environmental and climatic heterogeneity and strong vertical zonation of the forest vegetation.

## Introduction

Climate is changing more rapidly than ever: By 2020, global surface temperature has increased by 1°C relative to the mean of the years 1951–1980 [1]. Global warming is likely to reach 1.5°C between 2030 and 2052 if it will be continuing to increase at the current rate [2]. Ongoing climate warming is already causing shifts in species phenology, physiological and behavioural traits, geographical ranges, productivity, and disruption of species interactions [3–5]. Among terrestrial biomes, forests are most affected by ongoing global warming, as it not only impacts the survival rate of tree species, but also forces plants to cope with more frequently occurring extreme events such as severe droughts, floods, wildfires, etc. [6]. Since trees are the dominant species of forest ecosystems, any influence on them would cause cascading effects on the environment in terms of resource availability, local climate stability and ecosystem services [7]. Any impacts on tree species force dependent organisms to alter their life cycles and even cause their extinction in some cases [8]. Thus, understanding the fate of tree species in response to climate change is of crucial importance for conservation and management practices [4,9].

Broadleaf deciduous forests of Asia Minor occur adjacent to two major biodiversity hotspots, the Mediterranean Basin and the Caucasus [10]. The Euxine-Colchic broadleaf forests of Turkey and Georgia, and the Hyrcanian forests of northern Iran are among the most diverse temperate forest ecosystems in western Eurasia [11]. High landscape diversity in northern Turkey, Transcaucasia and northern Iran is buffering the effects of climate change and has created refugia for plant species during past glacial periods [12,13]. However, relatively little is known about how future climates will affect the distribution of broadleaf forests in these regions.

Beech (genus *Fagus*) is widely distributed across Eurasia, where it occurs in environments with sufficient rainfall and mild temperatures [14–17]. One of the most common broadleaf species of western Eurasia is the Oriental beech (*Fagus orientalis* Lipsky), which plays an important role in forming pure or mixed beech-conifer forests. This species occupies a narrow ecological niche being sensitive to late spring frosts and summer drought [18]. Consequently, it has been identified as especially vulnerable to climate change effects [19–21]. Since *F*. *orientalis* acts as a keystone species in its habitat and serves as an important resource to ecosystem services such as plywood, particleboard, furniture, flooring veneer, mining poles, railway tiles, paper, and firewood [22,23], it is of paramount importance to understand the fate of this species under climate change.

The common approach to investigate the impacts of climate change on plant distributions is using species distribution models (SDMs) [24,25]. These models help to identify regions in an area with changing environmental variables that have similar conditions to localities where the species has been recorded. By using occurrence (presence/absence) and abiotic environmental data, SDMs are frequently used as a tool for estimating the extent of a species’ range in the future or in the past [25,26]. Although a large number of authors have applied SDM to European woody species extensively in order to understand past and future distributions (e.g. [24–30]), there is a paucity of studies focused on Minor Asian tree taxa (e.g. [31,32]). Likewise, a great number of palaeobotanical studies investigated the late Quaternary history of *Fagus sylvatica* L. in Europe (e.g. [33–36]), and these studies were integrated with phylogeographic investigations (e.g. [37]).

In this study, we investigated the potential distribution of the Oriental beech in the past and in the future. To that end, we applied five SDM algorithms provided with the R-package `biomod2`to model potential distributions of this species under the present (1960–1990), past [Last Glacial Maximum (LGM), 21 ka, and mid-Holocene (MH), 6 ka], and future (Representative Concentration Pathway (RCP) 2.6, 4.5 and 8.5 for 2050 and 2070) climatic conditions obtained from MIROC-ESM and CCSM4 global climate models. Specifically, we pursue the following objectives: **(*i*)** to model the present distribution of *F*. *orientalis*, **(*ii*)** to reconstruct potential late Quaternary refugia of *F*. *orientalis*, **(*iii*)** to investigate the late Quaternary history of *Fagus* based on published palynological data, and finally, **(*iv*)** to project the future distribution of *F*. *orientalis* under different climate change scenarios.

## Materials and methods

### Study region and occurrence data

**Fig 1:**
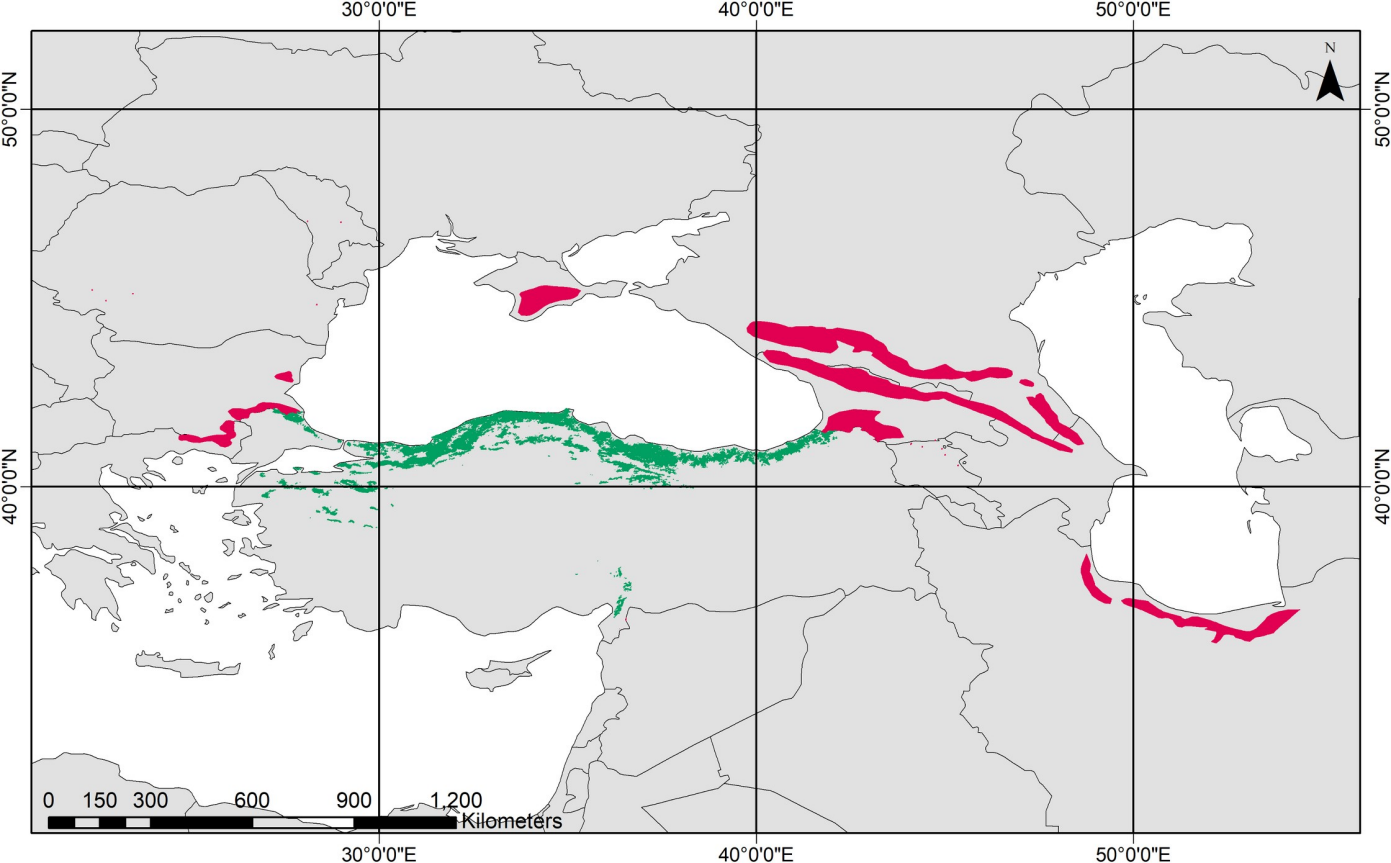
Modern range of *F*. *orientalis* based on occurrence data from EUFORGEN (red) and General Directorate of Forestry of Turkey (green).

The study region (22°–54° E 35°–47° N) is defined by the observed distribution range of *F*. *orientalis* with some extensions for possible future or past range expansions. Occurrence data were obtained from forest management plans of the General Directorate of Forestry of Turkey (GDF) for the distribution of the species in Turkey and the European Forest Genetic Resources Program (*F*. *orientalis*–EUFORGEN [38]) for the remaining areas. We merged these two layers of data in ArcGIS (version 10.3.1) and created a single, presence-only dataset (Fig 1) to be used in the models. All occurrence points, obtained from EUFORGEN and GDF, were transformed into 2.5ˊ spatial resolution using ArcGIS, generating 10,493 presence points for 457,681 raster cells across the study area.

### Environmental parameters

Since the climate is the main abiotic factor shaping species distribution [24], we used nine of 19 bioclimatic variables obtained from WorldClim version 1.4 [39] as the main environmental parameters (see S1 Table). The WorldClim dataset offers global climate model (GCM) simulations for the past (LGM and MH) and future (2050 and 2070) climatic conditions. We used MIROC-ESM and CCSM4 global climate simulations since they were the only two GCMs that provided data for all focal periods [40,41]. In addition, for the future simulations, climate scenarios based on IPCC reports were used [2]. According to these scenarios, in 2100, the expected CO_2_ levels will be 450 ppm, 650 ppm, and 1350 ppm in optimistic, moderate, and pessimistic scenarios, respectively. In addition, the expected increase in mean annual temperature will be 0.2–1.8°C, 1.0–2.6°C, and 2.6–4.8°C, in optimistic, moderate, and pessimistic scenarios, respectively [2]. To avoid issues arising from predictor collinearity, we retained only those bioclimatic variables with low correlation (|r| < 0.85) (Table 1, [42,43]). As a result, we used a subset consisting of nine bioclimatic variables (Table 2). In addition, the particular ecology of beech forests was taken into account when reducing the collinearity within the environmental parameters. According to Salamon-Albert et al. [44], temperature variables (annual mean, warmest, and coldest seasonal values) determine the distribution of beech forests. For Oriental beech forests at the latitudes covering our study region both temperature and precipitation factors play important roles. In addition, accounting for both temperature and precipitation in the model structure is important to consider water and moisture availability [45].

**Table 1:** Correlation matrix between 19 bioclimatic variables.

VARIABLES	BIO1	BIO10	BIO11	BIO12	BIO13	BIO14	BIO15	BIO16	BIO17	BIO18	BIO19	BIO2	BIO3	BIO4	BIO5	BIO6	BIO7	BIO8	BIO9
**BIO1**	1,00																		
**BIO10**	0,97	1,00																	
**BIO11**	0,96	0,87	1,00																
**BIO12**	-0,45	-0,56	-0,29	1,00															
**BIO13**	-0,59	-0,65	-0,46	0,90	1,00														
**BIO14**	-0,21	-0,32	-0,06	0,86	0,60	1,00													
**BIO15**	-0,17	-0,04	-0,31	-0,46	-0,10	-0,77	1,00												
**BIO16**	-0,59	-0,66	-0,46	0,91	0,99	0,60	-0,10	1,00											
**BIO17**	-0,21	-0,32	-0,05	0,87	0,60	0,99	-0,78	0,61	1,00										
**BIO18**	-0,68	-0,68	-0,61	0,82	0,87	0,61	-0,17	0,87	0,61	1,00									
**BIO19**	0,26	0,08	0,44	0,53	0,30	0,64	-0,61	0,30	0,64	-0,02	1,00								
**BIO2**	-0,09	0,00	-0,22	-0,32	-0,25	-0,34	0,50	-0,24	-0,36	-0,23	-0,28	1,00							
**BIO3**	-0,19	-0,29	-0,07	0,23	0,19	0,15	0,05	0,20	0,14	0,01	0,31	0,62	1,00						
**BIO4**	0,19	0,42	-0,09	-0,61	-0,47	-0,55	0,50	-0,47	-0,56	-0,25	-0,64	0,41	-0,44	1,00					
**BIO5**	0,93	0,98	0,80	-0,63	-0,71	-0,40	0,06	-0,71	-0,41	-0,74	0,02	0,20	-0,18	0,49	1,00				
**BIO6**	0,92	0,81	0,99	-0,20	-0,38	0,01	-0,38	-0,38	0,02	-0,54	0,49	-0,37	-0,15	-0,18	0,72	1,00			
**BIO7**	0,03	0,24	-0,23	-0,58	-0,45	-0,56	0,59	-0,45	-0,57	-0,28	-0,62	0,75	-0,05	0,90	0,39	-0,36	1,00		
**BIO8**	-0,15	-0,08	-0,23	0,25	0,33	0,11	0,11	0,32	0,12	0,51	-0,36	-0,08	-0,25	0,25	-0,11	-0,21	0,13	1,00	
**BIO9**	0,81	0,75	0,81	-0,56	-0,67	-0,37	-0,01	-0,66	-0,37	-0,87	0,31	0,14	0,13	0,03	0,78	0,75	0,04	-0,51	1,00

The bioclimatic variables selected for the present study are highlighted in red.

**Table 2:** Bioclimatic variables (precomputed in WorldClim dataset) used as environmental input in the models.

Abbreviations	Bioclimatic variables	Unit
**BIO1**	Annual Mean Temperature	°C
**BIO2**	Mean Diurnal Range	°C
**BIO3**	Isothermality	-
**BIO4**	Temperature Seasonality	°C
**BIO8**	Mean Temperature of Wettest Quarter	°C
**BIO9**	Mean Temperature of Driest Quarter	°C
**BIO12**	Annual Precipitation	mm
**BIO15**	Precipitation Seasonality	mm
**BIO19**	Precipitation of Coldest Quarter	mm

### Model structure and analysis

SDMs can describe or predict the probability of the presence or absence of a species across environmental gradients or in a specified geographical area [46]. We used the R-package ‘biomod2’ version 3.3–7.1 [47] to create potential-distribution maps of *F*. *orientalis*. To evaluate model performance, we implemented five different algorithms commonly used for SDMs provided with ‘biomod2’: Generalized linear model (GLM), general additive model (GAM), random forest (RF), BIOCLIM, and maximum entropy (MaxEnt). MaxEnt uses presence-only data. In contrast, GLM, GAM, and RF require both presence and absence data, and BIOCLIM uses pseudoabsence data [47], thus, we introduced 20,796 random background points to increase precision, as these add uniformity to the model while still subjected to climatic constraints [45]. Default settings were used for data formatting; 70% of the input data was used as a training sample. The related code is provided in the supplementary (S1 File).

Past and future climates were projected through the trained model, which is based on present conditions with observed distribution data. To evaluate and compare the model performances we used the Receiver Operating Characteristic Curve (Area Under the Curve, AUC) which takes values between 0 and 1 (AUC > 0.5 indicating that the model performed better than random), higher AUC value meaning better model performance [48]. Model outputs are given as the species’ presence probability in all algorithms.

To display and evaluate possible distributions we used both lowest predicted value [49] and sensitivity–specificity equality [50] methods to set a threshold for the minimum predicted value of observed occurrences, which was calculated as 0.585. We aimed at zero omission by setting this threshold and tried to balance sensitivity and specificity for increased accuracy.

### Fossil pollen data

To compare and test model performances for past projections we compiled published palynological records of *Fagus* comprising LGM and MH for the range of *F*. *orientalis*. Although *Fagus* pollen had low values in several pollen diagrams pointing to extra-local origin of the mother plant (e.g. [51]), we accepted such records as evidence for presence of oriental beech in the region during target periods (LGM, MH).

### Phylogeographic and taxonomic framework and inferred glacial refugia

In general, populations of *F*. *orientalis* are markedly more differentiated than *F*. *sylvatica* L. with F_ST_ (coefficient of differentiation among populations) being 0.157 in the former and 0.032 in the latter. In addition, much higher levels of allelic richness in this species indicating that Pleistocene bottlenecks did not deplete the gene pool to the extent they did in the European *F*. *sylvatica* [52]. Geographical subgroups of *F*. *orientalis* are morphologically and genetically highly distinct [52–56]. Populations ranging from eastern Bulgaria to northern Turkey and the Amanos Mountains form one cluster. Two additional highly distinct clusters involve the Caucasian populations on the one hand and the populations south of the Caspian Sea on the other hand. In contrast, the European *F*. *sylvatica* forms a single cluster [52]. In a recent study, Gömöry et al. [56] tested speciation scenarios in subgroups of *F*. *orientalis* under an approximate Bayesian framework. The number of generations was used to estimate divergence times between genetically distinct regional populations. Divergence times suggest that European populations of *F*. *sylvatica* diverged from Asian Minor populations (*F*. *orientalis*) at c. 1.222–0.7 Ma. The Crimean beech is a hybrid between Caucasian *F*. *orientalis* and *F*. *sylvatica*. It might have originated at around the Eemian interglacial (130–114 ka). Differentiation among the eastern populations happened much earlier: Caspian populations become isolated from Caucasian ones at 2.2–1.6 Ma, and from Turkish ones at 1.9–1.8 Ma. Finally, genetic isolation between populations of *F*. *sylvatica* from the Balkans and Central Europe and Apennine occurred much later, at 100 ka and 70 ka. This has important implications for inferring Pleistocene refugia for beech in western Eurasia. Essentially, when considering LGM and MH, *in situ* refugia must have existed for all geographical subgroups of *F*. *orientalis* because these regional groups had been isolated long before these events.

## Results

### Model evaluation and environmental parameters relevant for *Fagus orientalis* growth in Asia Minor

The results are indicating that models were good in distinguishing presence data from the background based on their AUC values varying from 0.79 to 0.99 (all > 0.7, Table 3, [57]). According to this, the best-fit algorithm was RF and the worst-fit was BIOCLIM. In the following, we will focus on the RF algorithm provided with ‘biomod2’, which fitted best for *F*. *orientalis*. Successful model results from the present climate could be used to identify and evaluate optimal environmental conditions for *F*. *orientalis* growth. The projections for the past, MH and LGM, and for the future, 2050 and 2070, gave different distributions with different climate change scenarios and global climate models (S1–S10 Figs).

**Table 3:** AUC_test_ values of all the algorithms, bioclim, maximum entropy (MaxEnt), generalized additive model (GAM), generalized linear model (GLM), and random forest (RF) performed with present climate conditions (1960–1990).

Algorithm	BIOCLIM	MaxEnt	GAM	GLM	RF
**AUC**_**test**_	0.79	0.94	0.96	0.93	0.99

### Past projections

**Fig 2:**
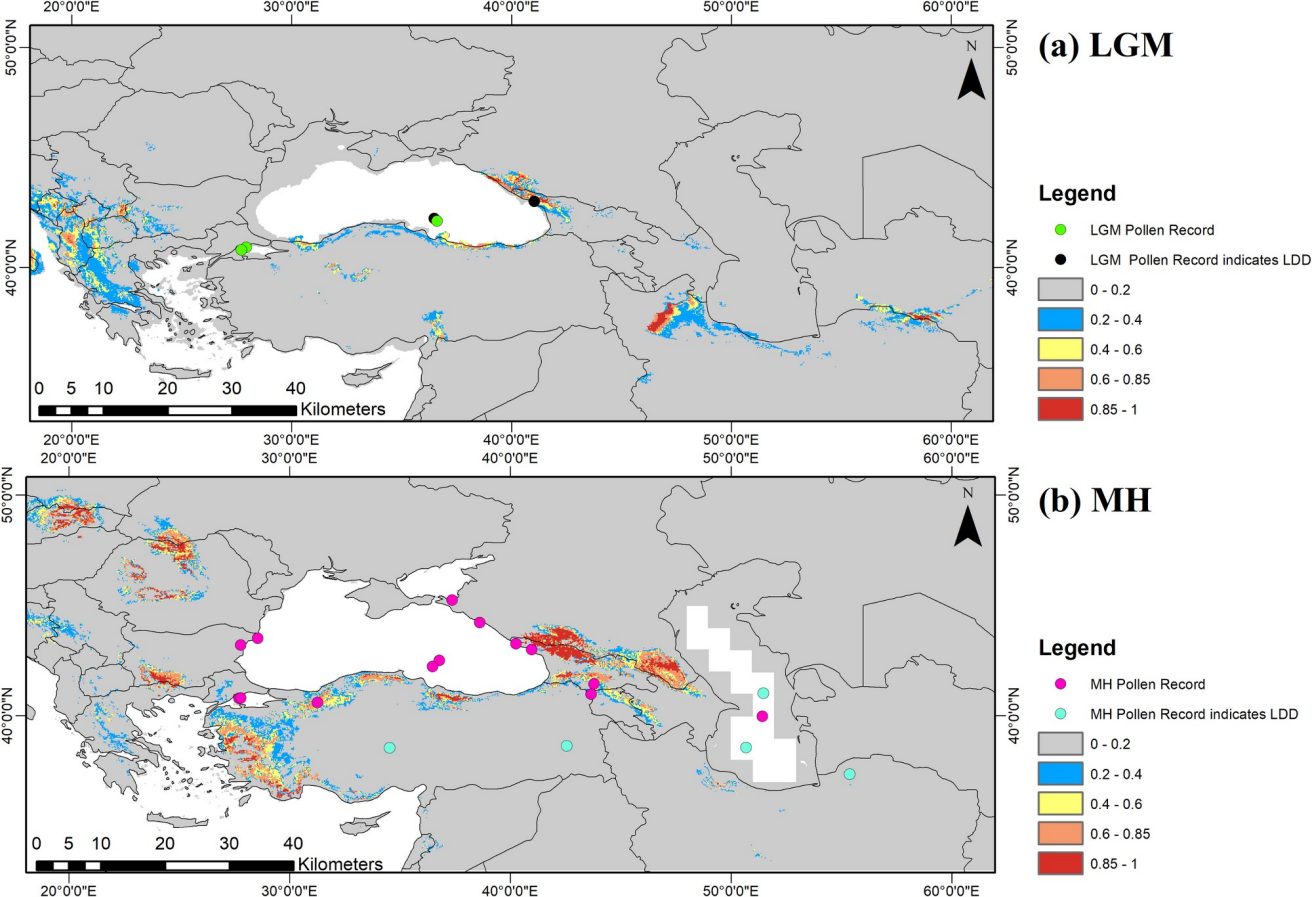
Past range projections with random forest algorithm; here only MIROC-ESM is shown (for CCSM4 see S8 Fig). Occurrence probability of the species is increasing from grey to red (absence to presence) for a) LGM and b) MH. Dots are indicating pollen records (Table 4).

Projections for the LGM at 21 ka and the MH at 6 ka from two different global climate models gave consistent results. Past projection outputs of RF are shown with the pollen records collected from published palynological studies of *Fagus* during LGM and MH (Fig 2). The model outputs show that the possible distribution of *Fagus* was mainly from the mid-Black Sea region to Caucasia and northern Iran to southern Turkmenistan during the LGM (Fig 2A). Additionally, RF predicted a refuge area in the Amanos region during the LGM. For the MH, the populations ranging from the mid-Black Sea region to Caucasia remained in these regions, whereas, the Iranian/Turkmen region almost lost its population, except some patches. During the MH, species distribution shifted towards the west, especially towards Europe and western Anatolia (Fig 2B). Outputs arising from other algorithms are shown in S1–S10 Figs.

### Late Quaternary palynological records of *Fagus* in Asia Minor

A general feature of the 30 pollen profiles containing *Fagus* is the sparse occurrence or absence of this genus during the LGM and its more or less continuous presence since about 10 ka (Fig 2 and Table 4). LGM values are usually low indicating long distance dispersal (LDD). One exception is the profile from the Sea of Marmara (core MD01-2430) that records beech with moderate abundance during the LGM. No suitable sediments recording the plant cover during the LGM are known from the southern Caspian area. This might be due to the marked drop in sea level (-50 m) of the Caspian Sea during this period [82]. In contrast, MH distribution of *F*. *orientalis* was continuous and abundant throughout its present range from eastern Bulgaria, the Sea of Marmara, northern Turkey, Crimea, the western Caucasus, and the southern Caspian Sea region. Percentages commonly are way above the proposed threshold values indicating local presence of *Fagus* reported in Lisitsyna et al. [51]. Moreover, we did not find convincing evidence for the presence of beech during MH outside its present distribution range. Two pollen profiles from Cappadocia and Lake Van have sporadic occurrences of *Fagus* pollen but these are very few pollen grains and most likely indicate LDD. This is also suggested by the overall composition of these pollen floras (open oak woodland in the Acıgöl area of Cappadocia [69], forest steppe in the Lake Van area [70]).

**Table 4:** Palaeobotanical records for the LGM and MH [58–81].

Country	Locality/site	LGM	MH	Pollen record	*Fagus* presence
Bulgaria	Black Sea Core GGC-18	No data	**Present**	12 ka	Cont. since 11 ka
Bulgaria	Varna Lake/Core 3	No data	**Present**	8 ka	Continuous
Bulgaria	W Black Sea	No data	**Present**	12 ka	Increase at c. 7 ka
Greece	Lesbos/Megali Limni 01	Absent	Absent	62–22 ka	Present at c. 32 ka, LDD
NW Turkey	Marmaris/MAR94-5	**Present**	Hiatus	30 ka	Continuous
NW Turkey	Marmaris/Core MD01-2430	**Present**	**Present**	23 ka	Continuous
NW Turkey	Marmaris/MAR98-12	No data	**Present**	c. 17 ka	Peak at HM
NW Turkey	Marmaris/MAR98-13	No data	**Present**	c. 18 ka	Peak at HM
NW Turkey	Black Sea/Core B-7	No data	**Present**	c. 12 ka	Peak at HM
NC Turkey	Yeniçağa, Bolu	No data	**Present**	c. 12 ka	Increase since c. 7 ka
NC Turkey	Abant Gölü, Bolu	No data	**Present**	c. 10 ka	Increase since c. 10 ka
NC Turkey	Black Sea/Core 22-GC3	**Present, LDD**	**Present**	18 ka	Cont., rapid increase at 8.5 ka
NC Turkey	Black Sea/Core 22-GC3/8	No data	No data	134–119 ka	Cont., rapid increase at 126 ka
NC Turkey	Black Sea/Core 25-GC1	**Present**	No data	63–19 ka	No specific information
C Turkey	Cappadocica/Eski Acıgöl I, II	No data	**Present, LDD**	15.6 ka	Sporadic, LDD
SE Turkey	Söğütlü, Van	No data	Absent	7 ka	Sporadic since c. 5 ka, LDD
SE Turkey	Lake Van/Core 90–04	No data	**Present, LDD**	12 ka	Sporadic since 10 ka, LDD
Georgia	Sukhumi/ Core no 723	No data	**Present**	c. 10 ka	Continuous
Georgia	Sukhumi/Dziguta Core 1	**Present, LDD**	No data	48–9 ka	Increase at c. 13 ka
Georgia	NE Black Sea/Core Ak-2575	No data	**Present**	10 ka	Continuous
Georgia	E Black Sea/Gagra, Core no 471	No data	**Present**	10 ka	Continuous
Georgia	Various sites	**Present**	No data	18±2 ka	No information
Iran	Lake Urmia	Absent	Absent	200 ka	Absent
Iran	Caspian Sea/Core CP14	No data	**Present**	5 ka	Continuous
Iran	Caspian Sea/Core GS18	No data	**Present, LDD**	12–2 ka	Cont. low, LDD
Iran	Caspian Sea/Core GS05	No data	**Present, LDD**	15 ka	Sporadic since c. 9 ka, LDD
NE Iran	Kongor Lake	No data	**Present, LDD**	6 ka	Discont. low, LDD

### Future projections

**Fig 3:**
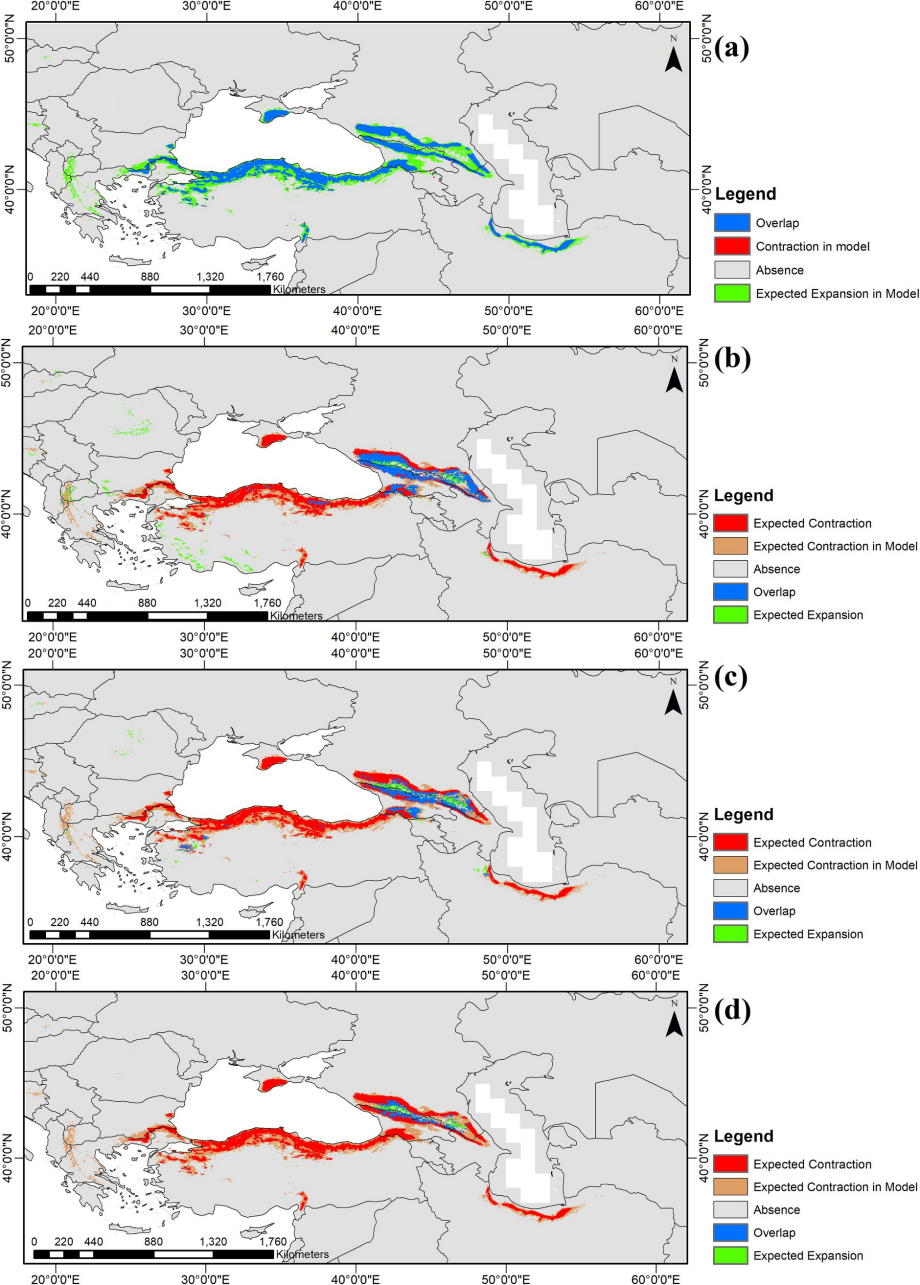
**Distribution maps of present and future projections of *F*. *orientalis* are shown from the random forests outputs of a) present, b) optimistic future scenario RCP 2.6, c) moderate future scenario RCP 4.5, and d) pessimistic future scenario RCP 8.5.** Red areas show the contraction of species occurrence based on observed data and future projections, brown areas show the contraction of species occurrence based on present and future projections, blue areas represent overlap of species occurrence based on observed data and present projection, green areas show the expansion of species occurrence based on observed data and projections. We quantified the cell numbers for each category in Table 5. Spatial resolution: 2.5ˊ.

The combined results from both global climate models with different scenarios show that in the future, the main geographical shift of *F*. *orientalis* will be towards the northeast of its present distribution. According to the RF model, a severe range contraction of the species is expected in relation to the present distribution provided by the projected model and the compiled occurrence data (Table 5 and Fig 1). In addition, a refuge area was projected southwest of the Caspian Sea including the southwestern Caspian coastal area and coastal mountains (Fig 3, for additional algorithms see S1–S10 Figs). The proportion of species presence in the RF MIROC-ESM projections from different scenarios is shown in Table 5. According to the table, the proportion of presence cells is 1.49% in the optimistic, 0.95% in the moderate, and 0.42% in the pessimistic climate scenario. Details of the spatial changes are shown in Fig 3.

**Table 5:** Spatial cell numbers of contraction, overlap, and expansion shown in Fig 3 are quantified in this table.

Period	Future Scenario	Expected Contraction	Expected Contraction in model	Overlap	Expected Expansion	Overlap with observed occurrence data (%)
**Present**	–	115	–	10,372	9.444	98.85
**2070**	RCP 2.6	7,944	7919	3,954	1.558	37.68
	RCP 4.5	9,009	8226	2,582	927	24.61
	RCP 8.5	9,816	8914	1,087	446	10.36

Overlap of the species occurrence between the observed data and model outputs are given as percentages. In future projections, from optimistic to pessimistic scenario, contractions are increasing, overlap and expansion are decreasing.

## Discussion

### Model evaluation for different SDM algorithms

Modelling the spatial distribution of *F*. *orientalis* provided details of the species responses to different climate change scenarios and identified possible past refugia by means of projected past distributions and palynological data. A key assumption of SDM with bioclimatic variables is that the ranges of the modelled species are in equilibrium with climate [25, 29, 83]. In general, the modelled present distributions fitted reasonably well with the observed distribution data, which allowed us to use the trained model for past and future simulations. As mentioned before, all models ran successfully according to their AUC values (Table 3), and the combination of regression and classification approaches, as well as the in-algorithm verification process provided more accurate outcomes in RF and made this algorithm the most successful one [84]. On the other hand, according to the AUC values (Table 3) the least successful algorithm was BIOCLIM. Due to the advanced characteristics and calculations of other algorithms, it was expected that they would give a better fit than BIOCLIM. One problematic algorithm was GAM (S5 and S6 Figs) with a very wide distribution of Oriental beech projected for LGM and MH. Although GAM has the second-best AUC value, we interpreted this as an overfitted distribution. It is known that this algorithm is highly sensitive to large sample size since the fitted functions are not constrained to any functional form when sample size increases [85]. Further, it is noteworthy that while there are substantial differences between modelling performances from different algorithms, the outcomes are consistent, which is a hint for repeatability [86].

### The present distribution of Oriental beech

The present distribution of *Fagus orientalis* in the RF algorithm/MIROC-ESM was largely congruent with the observed presence records (Figs 1 and 3A), which is also illustrated by its high AUC value. A range expansion in the European region (Fig 3A) most probably reflects similar present growing conditions of *F*. *orientalis* and its sister species *F*. *sylvatica*. Hence, it is not surprising that models recognize habitats of *F*. *sylvatica* as suitable for *F*. *orientalis* [8, 17]. Although there is a close match between observed and modelled distribution, it is interesting to note that the model predicted range expansion to the south of the Pontic Mountains (Kuzey Anadolu Dağları). Under present conditions, this is unlikely as the rain shadow south of the main ridge of the Pontic Mountains creates a sharp change from fully humid *Cf* climates (see [16, 87]) to Mediterranean *Cs* and continental, arid *BS* climates. Whereas, *Fagus* is replaced by Pinaceae at higher elevations, it is replaced by *Quercus* species (e.g. *Quercus macranthera* Fisch. et Mey.) both at higher elevations and in drier areas beyond the main ridge [88].

The situation is similar in the Iranian Alborz Mountains, where *Quercus macranthera* replaces *Fagus* at higher elevations [89]. The Amanos Mountains are distinctive, because Oriental beech is confined to higher elevations under a distinct microclimate, thus, it is remarkable that the model could identify this relict population here [90].

### Past reconstructions and palynological data comparison

Probability estimates for occurrences of Oriental beech during the LGM by ‘biomod2’ (Fig 2A) are to some extent congruent with known pollen records. Unambiguous occurrences of beech pollen from LGM deposits are known from the Sea of Marmara, off the central northern Turkish Black Sea coast, and from Sukhumi. These occurrences coincide with highly probable model estimates (red in Fig 2A). While estimates on presence in Transcaucasia and southwest of the Caspian Sea are sensible although not supported by palynological data, a refuge area in Turkmenistan at 60°E east of the Caspian Sea is highly unrealistic in view of the present arid climate in this region. During the LGM, a cold continental climate would not have provided suitable conditions for the growth of beech. ‘biomod2’ reconstructed a further refugium in the Alborz Mountains south of 35°N. Although no pollen record is available for this region, the Amanos Mountains with a high relief, deep valleys, and slopes facing the sea likely provided a refuge for beech populations during the LGM.

The core region of *F*. *orientalis* estimated for the MH (Fig 2B) includes the Turkish Black Sea Coast and the Caucasus. This is also suggested by unambiguous pollen records known from this area (Fig 2B and Table 4). In contrast, ‘biomod2’ with RF predicted a rather limited distribution south of the Caspian Sea, from where abundant pollen records of beech are known as well. Palynological data and paleoclimate modelling [91, 92] suggest that temperate regions during the MH might have been warmer than today during summer and colder during winter because of astronomical forcing. Today, fully humid, warm temperate *Cf* climates are much less common along the southern Caspian Sea coast than south of the Black Sea and along the northern and southern foothills of the Caucasus ([16]; http://koeppen-geiger.vu-wien.ac.at/kmz/Global_1986–2010_KG_5m.kmz.zip provided by Veterinary University of Vienna, http://koeppen-geiger.vu-wien.ac.at/present.htm). In northern Iran, a stronger Mediterranean influence is expressed by lower summer precipitation and higher summer temperatures (http://koeppen-geiger.vu-wien.ac.at/kmz/Global_1986–2010_KG_5m.kmz.zip). Hence, warmer summers during the Mid Holocene would have increased this Mediterranean influence and resulted in a much-reduced distribution of Oriental beech in the models. As today, these unfavourable conditions might have been compensated by high air moisture and a sky covered with fog for most of the year and particularly during the growing season [89]. However, the predicted presence of beech in the western corner of the Caspian coast makes sense, as this area–constrained by wind directions and high mountains to the south and a large water body to the north, very likely could have supported growth of beech during the MH (Fig 2B).

### Future projections

Future projections show that *F*. *orientalis* will face a dramatic range contraction in the future (Figs 3B–3D and S1–S10). The projections suggest that the species will mainly thrive in the Caucasus region. Since it is assumed that future climatic conditions will cause an increase in temperature and drought, the mountainous regions of Caucasia will be preferred by Oriental beech for possibly more suitable temperature and moisture conditions. The RF algorithm suggests a slight expansion south of the Caspian Sea, along the southwestern Caspian coast. This supports the idea of potential refugia for *F*. *orientalis* in the future in this region and emphasizes the importance of this region as a long-term refugium for temperate tree species. Both regions are characterized by high mountain ranges. The Alborz Mountains, located just south of the Caspian Sea, providing a barrier for humid air masses thereby creating warm and humid conditions from sea level to high elevations. This leads to a large number of suitable habitats under a changing climate as plants can move vertically. A similar situation is met in the western parts of Caucasia where humid air masses from the Black Sea create markedly humid conditions along the coast and on the slopes of the greater and lesser Caucasus (e.g [93]). Towards the east, climatic conditions become drier, but the great vertical gradient remains, thus providing a dynamic environment for tree species to respond to changing environments by vertical movements. A recent study by Martin-Benito et al. [21] supports the notion that due to little drought response and positive effects of spring-summer warmth, mid- to high-elevation sites in the Caucasus might be potential major climatic refugia for *Fagus orientalis* in the future.

### Comparison with SDMs of European beech (*Fagus sylvatica*)

Process-based and statistical modelling approaches have been widely used for modelling past and future distribution of a close relative of *F*. *orientalis*, *Fagus sylvatica*. For instance, Gisecke et al. [27] used the process-based bioclimatic model to understand the distribution of *F*. *sylvatica* during the MH. In agreement with our findings, they also compared their model results with pollen records and found some mismatches, in particular in the northern and western parts of the distribution. They emphasized the need for testing models against palynological data to increase their predictive value. Likewise, a recent study modelling the modern and past distribution of deciduous oaks (*Quercus robur* L.) also showed a mismatch between the modelled LGM distribution and the one inferred from pollen data [32]. This study suggested that the more restricted distribution data from the pollen record might be due to competition of deciduous oaks with pines and Mediterranean evergreen oaks. Saltré et al. [94] applied a process-based approach coupled with migration models to assess whether the present distribution of *F*. *sylvatica* is controlled more by climatic conditions or the migration ability during the MH in Europe. Their findings suggested that the northeast boundary of *F*. *sylvatica* distribution is limited by climatic conditions whereas the north-west boundary is limited by migration ability. This is very similar to the situation in *F*. *orientalis*, where, clearly, the (north)eastern boundary is controlled by climate whereas the western boundary is limited by migration ability. However, in contrast to *F*. *sylvatica*, the westward expansion of Oriental beech is limited because it is replaced towards the west by its sister species *F*. *sylvatica*. Kramer et al. [95] investigated *F*. *sylvatica* distribution under climate change with both process-based and statistical modelling approaches. They found that statistical species distribution models exhibited a better goodness-of-fit for future projections, with the southern limit of the *F*. *sylvatica* distribution shifted towards the north. On the other hand, future projections of Dyderski et al. [8] for twelve European forest tree species including European beech suggested that the distribution ranges in western and southern Europe will decrease. This seems plausible in view of the predicted climate change towards summer-dry *Cs* climates in western and southwestern Europe during 2070–2100 ([96]; Global_Shift_A1FI_1976–2100_30m.kmz).

## Conclusions

In this study, we investigated the late Quaternary history of *F*. *orientalis* utilizing palynological data and past reconstructions and estimated the future distribution of the species in response to global climate change. We compared model outputs to the observed present distribution of *F*. *orientalis* and palynological records from LGM and MH. Based on these empirical data, the RF algorithm, among five algorithms in the R-package ‘biomod2’, performed best with more plausible results for both past and future distributions. As in previous studies using *F*. *sylvatica* and *Quercus robur* as focal species, models tend to overestimate occurrence probabilities. For LGM and MH, the results were roughly in accord with palynological data for the Sea of Marmara and the Black Sea region, while palynological data did not support potential refugia south of the Caspian Sea during LGM. However, genetically long isolated populations of Oriental beech south of the Caspian Sea strongly suggest that they survived the LGM in-situ. For future projections, with continuing climate change, *F*. *orientalis* populations in the western Eurasian region will be decreasing with increasing drought; the geographical range will contract leaving only two possible refugia, the southern Caspian Sea region (northern Iran) and the Caucasus.

This study shows, albeit they can be improved by including other environmental data (e.g. aspect, soil), that our models provide crucial information on the effect of climate change on *F*. *orientalis* in Asia Minor even when only considering the climate. In addition, these results emphasize the importance of combining evidence from modelling with real-world data, such as the rich Quaternary pollen record of *Fagus*, to understand species-specific responses to changing environments. Hence, these results should be taken into account in order to improve conservation and management plans for this important forest species in western Eurasia.

## Supporting information

S1 Table 19 bioclimatic variables obtained from WorldClim version 1.4. (PDF)

S1 File R code sheet for biomod2 package to perform SDM. (PDF)

S1 Fig Probability distributions of *F*. *orientalis* predicted for the present, MH, LGM, pessimistic (RCP 8.5) 2050 and 2070, moderate (RCP 4.5) 2050 and 2070, and optimistic (RCP 2.6) 2050 and 2070 with MaxEnt algorithm and MIROC–ESM GCM. (TIF)

S2 Fig Probability distributions of *F*. *orientalis* predicted for the present, MH, LGM, pessimistic (RCP 8.5) 2050 and 2070, moderate (RCP 4.5) 2050 and 2070, and optimistic (RCP 2.6) 2050 and 2070 with MaxEnt algorithm and CCSM4 GCM. (TIF)

S3 Fig Probability distributions of *F*. *orientalis* predicted for the present, MH, LGM, pessimistic (RCP 8.5) 2050 and 2070, moderate (RCP 4.5) 2050 and 2070, and optimistic (RCP 2.6) 2050 and 2070 with GLM algorithm and MIROC–ESM GCM. (TIF)

S4 Fig Probability distributions of *F*. *orientalis* predicted for the present, MH, LGM, pessimistic (RCP 8.5) 2050 and 2070, moderate (RCP 4.5) 2050 and 2070, and optimistic (RCP 2.6) 2050 and 2070 with GLM algorithm and CCSM4 GCM. (TIF)

S5 Fig The probability of *F*. *orientalis* predicted for the present, MH, LGM, pessimistic (RCP 8.5) 2050 and 2070, moderate (RCP 4.5) 2050 and 2070, and optimistic (RCP 2.6) 2050 and 2070 with GAM algorithm and MIROC GCM. (TIF)

S6 Fig The probability of *F*. *orientalis* predicted for the present, MH, LGM, pessimistic (RCP 8.5) 2050 and 2070, moderate (RCP 4.5) 2050 and 2070, and optimistic (RCP 2.6) 2050 and 2070 with GAM algorithm and CCSM4 GCM. (TIF)

S7 Fig Probability distributions of *F*. *orientalis* predicted for the present, MH, LGM, pessimistic (RCP 8.5) 2050 and 2070, moderate (RCP 4.5) 2050 and 2070, and optimistic (RCP 2.6) 2050 and 2070 with RF algorithm and MIROC–ESM GCM. (TIF)

S8 Fig Probability distributions of *F*. *orientalis* predicted for the present, MH, LGM, pessimistic (RCP 8.5) 2050 and 2070, moderate (RCP 4.5) 2050 and 2070, and optimistic (RCP 2.6) 2050 and 2070 with RF algorithm and CCSM4 GCM. (TIF)

S9 Fig Probability distributions of *F*. *orientalis* predicted for the present, MH, LGM, pessimistic (RCP 8.5) 2050 and 2070, moderate (RCP 4.5) 2050 and 2070, and optimistic (RCP 2.6) 2050 and 2070 with BIOCLIM algorithm and MIROC–ESM GCM. (TIF)

S10 Fig Probability distributions of *F*. *orientalis* predicted for the present, MH, LGM, pessimistic (RCP 8.5) 2050 and 2070, moderate (RCP 4.5) 2050 and 2070, and optimistic (RCP 2.6) 2050 and 2070 with BIOCLIM algorithm and CCSM4 GCM. (TIF)
