# Supplementary material for: Past, present and future distributions of Oriental beech (Fagus orientalis) under climate change projections
Source: PLoS One. 2020 Nov 17;15(11):e0242280. doi: 10.1371/journal.pone.0242280 (PMC7671530; doi:10.1371/journal.pone.0242280)
Supplement: S1 Table — (PDF) [file pone.0242280.s001.pdf]

S1 Table. 19 bioclimatic variables obtained from WorldClim version 1.4.

Abbreviation	Bioclimatic variables
BIO1	Annual Mean Temperature
BIO2	Mean Diurnal Range (Mean of monthly (max temp - min temp))
BIO3	Isothermality (BIO2/BIO7) (* 100)
BIO4	Temperature Seasonality (standard deviation *100)
BIO5	Max Temperature of Warmest Month
BIO6	Min Temperature of Coldest Month
BIO7	Temperature Annual Range (BIO5-BIO6)
BIO8	Mean Temperature of Wettest Quarter
BIO9	Mean Temperature of Driest Quarter
BIO10	Mean Temperature of Warmest Quarter
BIO11	Mean Temperature of Coldest Quarter
BIO12	Annual Precipitation
BIO13	Precipitation of Wettest Month
BIO14	Precipitation of Driest Month
BIO15	Precipitation Seasonality (Coefficient of Variation)
BIO16	Precipitation of Wettest Quarter
BIO17	Precipitation of Driest Quarter
BIO18	Precipitation of Warmest Quarter
BIO19	Precipitation of Coldest Quarter
