# Supplementary material for: Past, present and future distributions of Oriental beech (Fagus orientalis) under climate change projections
Source: PLoS One. 2020 Nov 17;15(11):e0242280. doi: 10.1371/journal.pone.0242280 (PMC7671530; doi:10.1371/journal.pone.0242280)
Supplement: S1 File — (PDF) [file pone.0242280.s002.pdf]

S1 File. R code sheet for biomod2 package to perform SDM.

```
library(biomod2)

setwd("C:/Users/dilsa/Desktop")

#load the data

fagus <- read.csv("presence25.csv", header = TRUE, sep = ",")

head(fagus)

plot(fagus)


#getting presence values from data

myRespName <- 'Fagus_orientalis'

myResp <- as.numeric(fagus[,myRespName])

#getting presence coordinates fromd data

myRespXY <- fagus[,c("X", "Y")]


#environmental layers as explanatory variables


bio1 <- raster("C:/Users/dilsa/Desktop/current_2.5_tif/bio1.tif")

bio2 <- raster("C:/Users/dilsa/Desktop/current_2.5_tif/bio2.tif")

bio3 <- raster("C:/Users/dilsa/Desktop/current_2.5_tif/bio3.tif")

bio4 <- raster("C:/Users/dilsa/Desktop/current_2.5_tif/bio4.tif")

bio8 <- raster("C:/Users/dilsa/Desktop/current_2.5_tif/bio8.tif")

bio9 <- raster("C:/Users/dilsa/Desktop/current_2.5_tif/bio9.tif")

bio12 <- raster("C:/Users/dilsa/Desktop/current_2.5_tif/bio12.tif")
```

```
bio15 <- raster("C:/Users/dilsa/Desktop/current_2.5_tif/bio15.tif")

bio19 <- raster("C:/Users/dilsa/Desktop/current_2.5_tif/bio19.tif")


current_bios = stack(bio1, bio2, bio3, bio4, bio8, bio9, bio12, bio15, bio19)


myBiomodData <- BIOMOD_FormatingData(resp.var = myResp,
                                     expl.var = current_bios,
                                     resp.xy = myRespXY,
                                     resp.name = myRespName,
                                     PA.nb.rep = 1,
                                     PA.nb.absences = 20796,
                                     PA.strategy = 'random',
                                     na.rm = TRUE)


plot(myBiomodData)


#modeling


myBiomodOption <- BIOMOD_ModelingOptions()


myBiomodModelOut <- BIOMOD_Modeling(
  myBiomodData,
  models = c('GLM','GAM','RF','SRE','MAXENT.Phillips'),
```

```
  models.options = myBiomodOption,
  NbRunEval=3,
  DataSplit=70,
  Prevalence=0.5,
  VarImport=3,
  models.eval.meth = c('TSS','ROC'),
  SaveObj = TRUE,
  rescal.all.models = TRUE,
  do.full.models = FALSE,
  modeling.id = paste('Fagus orientalis',"FirstModeling",sep=""))

myBiomodModelOut

#get all models evaluation
myBiomodModelEval <- get_evaluations(myBiomodModelOut)
myBiomodModelEval
# print the dimnames of this object
dimnames(myBiomodModelEval)
# let's print the TSS scores
myBiomodModelEval["TSS","Testing.data","GLM",,]
myBiomodModelEval["TSS","Testing.data","GAM",,]
myBiomodModelEval["TSS","Testing.data","RF",,]
myBiomodModelEval["TSS","Testing.data","SRE",,]
```

```
myBiomodModelEval["TSS","Testing.data","MAXENT.Phillips",,]


# let's print the ROC scores of all selected models
myBiomodModelEval["ROC","Testing.data",,,]
# print variable importances
get_variables_importance(myBiomodModelOut)

# Ensemble Modeling
myBiomodEM <- BIOMOD_EnsembleModeling(
  modeling.output = myBiomodModelOut,
  chosen.models = 'all',
  em.by='all',
  eval.metric = c('ROC'),
  eval.metric.quality.threshold = c(0.7),
  prob.mean = T,
  prob.cv = T,
  prob.ci = T,
  prob.ci.alpha = 0.05,
  prob.median = T,
  committee.averaging = T,
  prob.mean.weight = T,
  prob.mean.weight.decay = 'proportional' )
```

```
# print summary

myBiomodEM

# get evaluation scores

EM <- get_evaluations(myBiomodEM)

EM

plot(myBiomodEM)




# Model Projection

# First let’s project the individual models on our current conditions

# (the globe) to visualize them.


# projection over the globe under current conditions

myBiomodProj <- BIOMOD_Projection(

  modeling.output = myBiomodModelOut,

  new.env = current_bios,

  proj.name = 'current',

  selected.models = 'all',

  binary.meth = 'TSS',

  compress = 'xz',

  clamping.mask = F,

  output.format = '.grd')

# summary of crated oject
```

```
myBiomodProj

# files created on hard drive

list.files("Fagus.orientalis/proj_current/")

# make some plots sub-selected by str.grep argument

plot(myBiomodProj, str.grep = 'RUN1_GLM')

plot(myBiomodProj, str.grep = 'RUN1_GAM')

plot(myBiomodProj, str.grep = 'RUN1_RF')

plot(myBiomodProj, str.grep = 'RUN1_SRE')

plot(myBiomodProj, str.grep = 'RUN1_MAXENT.Phillips')

# if you want to make custom plots, you can also get the projected map

myCurrentProj <- get_predictions(myBiomodProj)

myCurrentProj


##CLIMATE CHANGE PROJECTIONS


# MIROC #


#Last Glacial Maximum MIROC


a_bio1 <- raster("C:/Users/dilsa/Desktop/lgm_miroc_2.5_tif/bio1.tif")

a_bio2 <- raster("C:/Users/dilsa/Desktop/lgm_miroc_2.5_tif/bio2.tif")

a_bio3 <- raster("C:/Users/dilsa/Desktop/lgm_miroc_2.5_tif/bio3.tif")

a_bio4 <- raster("C:/Users/dilsa/Desktop/lgm_miroc_2.5_tif/bio4.tif")
```

```
a_bio8 <- raster("C:/Users/dilsa/Desktop/lgm_miroc_2.5_tif/bio8.tif")

a_bio9 <- raster("C:/Users/dilsa/Desktop/lgm_miroc_2.5_tif/bio9.tif")

a_bio12 <- raster("C:/Users/dilsa/Desktop/lgm_miroc_2.5_tif/bio12.tif")

a_bio15 <- raster("C:/Users/dilsa/Desktop/lgm_miroc_2.5_tif/bio15.tif")

a_bio19 <- raster("C:/Users/dilsa/Desktop/lgm_miroc_2.5_tif/bio19.tif")


a_bios = stack(a_bio1, a_bio2, a_bio3, a_bio4, a_bio8, a_bio9,

               a_bio12, a_bio15, a_bio19)




# projection under lgm_miroc conditions

LGM_MIROC_Proj <- BIOMOD_Projection(

  modeling.output = myBiomodModelOut,

  new.env = a_bios,

  proj.name = 'LGM_MIROC',

  selected.models = 'all',

  binary.meth = 'TSS',

  compress = 'xz',

  clamping.mask = F,

  output.format = '.grd')


# summary of crated object

LGM_MIROC_Proj
```

```
# files created on hard drive

list.files("Fagus.orientalis/proj_LGM_MIROC")

# make some plots sub-selected by str.grep argument

plot(LGM_MIROC_Proj, str.grep = 'RUN1_GLM')

plot(LGM_MIROC_Proj, str.grep = 'RUN1_GAM')

plot(LGM_MIROC_Proj, str.grep = 'RUN1_RF')

plot(LGM_MIROC_Proj, str.grep = 'RUN1_SRE')

plot(LGM_MIROC_Proj, str.grep = 'RUN1_MAXENT.Phillips')

# if you want to make custom plots, you can also get the projected map

myLGMMProj <- get_predictions(LGM_MIROC_Proj)

myLGMMProj


#Mid-Holocene MIROC


b_bio1 <- raster("C:/Users/dilsa/Desktop/mh_miroc_2.5_tif/bio1.tif")

b_bio2 <- raster("C:/Users/dilsa/Desktop/mh_miroc_2.5_tif/bio2.tif")

b_bio3 <- raster("C:/Users/dilsa/Desktop/mh_miroc_2.5_tif/bio3.tif")

b_bio4 <- raster("C:/Users/dilsa/Desktop/mh_miroc_2.5_tif/bio4.tif")

b_bio8 <- raster("C:/Users/dilsa/Desktop/mh_miroc_2.5_tif/bio8.tif")

b_bio9 <- raster("C:/Users/dilsa/Desktop/mh_miroc_2.5_tif/bio9.tif")

b_bio12 <- raster("C:/Users/dilsa/Desktop/mh_miroc_2.5_tif/bio12.tif")

b_bio15 <- raster("C:/Users/dilsa/Desktop/mh_miroc_2.5_tif/bio15.tif")
```

```
b_bio19 <- raster("C:/Users/dilsa/Desktop/mh_miroc_2.5_tif/bio19.tif")

b_bios = stack(b_bio1, b_bio2, b_bio3, b_bio4, b_bio8, b_bio9,
               b_bio12, b_bio15, b_bio19)

# projection under mh_miroc conditions
MH_MIROC_Proj <- BIOMOD_Projection(
  modeling.output = myBiomodModelOut,
  new.env = b_bios,
  proj.name = 'MH MIROC',
  selected.models = 'all',
  binary.meth = 'TSS',
  compress = 'xz',
  clamping.mask = F,
  output.format = '.grd')

# summary of crated object
MH_MIROC_Proj

# files created on hard drive
list.files("Fagus.orientalis/proj_MH MIROC")
# make some plots sub-selected by str.grep argument
```

```
plot(MH_MIROC_Proj, str.grep = 'RUN1_GLM')

plot(MH_MIROC_Proj, str.grep = 'RUN1_GAM')

plot(MH_MIROC_Proj, str.grep = 'RUN1_RF')

plot(MH_MIROC_Proj, str.grep = 'RUN1_SRE')

plot(MH_MIROC_Proj, str.grep = 'RUN1_MAXENT.Phillips')

# if you want to make custom plots, you can also get the projected map

myMHMProj <- get_predictions(MH_MIROC_Proj)

myMHMProj


#2050 MIROC


c_bio1 <- raster("C:/Users/dilsa/Desktop/2050_miroc_2.5_tif/bio1.tif")

c_bio2 <- raster("C:/Users/dilsa/Desktop/2050_miroc_2.5_tif/bio2.tif")

c_bio3 <- raster("C:/Users/dilsa/Desktop/2050_miroc_2.5_tif/bio3.tif")

c_bio4 <- raster("C:/Users/dilsa/Desktop/2050_miroc_2.5_tif/bio4.tif")

c_bio8 <- raster("C:/Users/dilsa/Desktop/2050_miroc_2.5_tif/bio8.tif")

c_bio9 <- raster("C:/Users/dilsa/Desktop/2050_miroc_2.5_tif/bio9.tif")

c_bio12 <- raster("C:/Users/dilsa/Desktop/2050_miroc_2.5_tif/bio12.tif")

c_bio15 <- raster("C:/Users/dilsa/Desktop/2050_miroc_2.5_tif/bio15.tif")

c_bio19 <- raster("C:/Users/dilsa/Desktop/2050_miroc_2.5_tif/bio19.tif")


c_bios = stack(c_bio1, c_bio2, c_bio3, c_bio4, c_bio8, c_bio9,

               c_bio12, c_bio15, c_bio19)
```

```
# projection under 2050_miroc conditions

future1_MIROC_Proj <- BIOMOD_Projection(

  modeling.output = myBiomodModelOut,

  new.env = c_bios,

  proj.name = '2050 MIROC',

  selected.models = 'all',

  binary.meth = 'TSS',

  compress = 'xz',

  clamping.mask = F,

  output.format = '.grd')


# summary of crated object

future1_MIROC_Proj


# files created on hard drive

list.files("Fagus.orientalis/proj_2050 MIROC")

# make some plots sub-selected by str.grep argument

plot(future1_MIROC_Proj, str.grep = 'RUN1_GLM')

plot(future1_MIROC_Proj, str.grep = 'RUN1_GAM')

plot(future1_MIROC_Proj, str.grep = 'RUN1_RF')

plot(future1_MIROC_Proj, str.grep = 'RUN1_SRE')

plot(future1_MIROC_Proj, str.grep = 'RUN1_MAXENT.Phillips')
```

```
# if you want to make custom plots, you can also get the projected map

my2050MProj <- get_predictions(future1_MIROC_Proj)

my2050MProj


#2070 MIROC


d_bio1 <- raster("C:/Users/dilsa/Desktop/2070_miroc_2.5_tif/bio1.tif")

d_bio2 <- raster("C:/Users/dilsa/Desktop/2070_miroc_2.5_tif/bio2.tif")

d_bio3 <- raster("C:/Users/dilsa/Desktop/2070_miroc_2.5_tif/bio3.tif")

d_bio4 <- raster("C:/Users/dilsa/Desktop/2070_miroc_2.5_tif/bio4.tif")

d_bio8 <- raster("C:/Users/dilsa/Desktop/2070_miroc_2.5_tif/bio8.tif")

d_bio9 <- raster("C:/Users/dilsa/Desktop/2070_miroc_2.5_tif/bio9.tif")

d_bio12 <- raster("C:/Users/dilsa/Desktop/2070_miroc_2.5_tif/bio12.tif")

d_bio15 <- raster("C:/Users/dilsa/Desktop/2070_miroc_2.5_tif/bio15.tif")

d_bio19 <- raster("C:/Users/dilsa/Desktop/2070_miroc_2.5_tif/bio19.tif")


d_bios = stack(d_bio1, d_bio2, d_bio3, d_bio4, d_bio8, d_bio9,

               d_bio12, d_bio15, d_bio19)


# projection under 2070_miroc conditions

future2_MIROC_Proj <- BIOMOD_Projection(

  modeling.output = myBiomodModelOut,

  new.env = d_bios,
```

```
  proj.name = '2070 MIROC',
  selected.models = 'all',
  binary.meth = 'TSS',
  compress = 'xz',
  clamping.mask = F,
  output.format = '.grd')
# summary of crated object
future2_MIROC_Proj
# files created on hard drive
list.files("Fagus.orientalis/proj_2070 MIROC")
# make some plots sub-selected by str.grep argument
plot(future2_MIROC_Proj, str.grep = 'RUN1_GLM')
plot(future2_MIROC_Proj, str.grep = 'RUN1_GAM')
plot(future2_MIROC_Proj, str.grep = 'RUN1_RF')
plot(future2_MIROC_Proj, str.grep = 'RUN1_SRE')
plot(future2_MIROC_Proj, str.grep = 'RUN1_MAXENT.Phillips')
# if you want to make custom plots, you can also get the projected map
my2070MProj <- get_predictions(future2_MIROC_Proj)
my2070Mproj
```

# Do this part again for also all climate conditions in the second GCM: CCSM4.
