# Supplementary figures and images for: Past, present and future distributions of Oriental beech (Fagus orientalis) under climate change projections
Source: PLoS One. 2020 Nov 17;15(11):e0242280. doi: 10.1371/journal.pone.0242280 (PMC7671530; doi:10.1371/journal.pone.0242280)

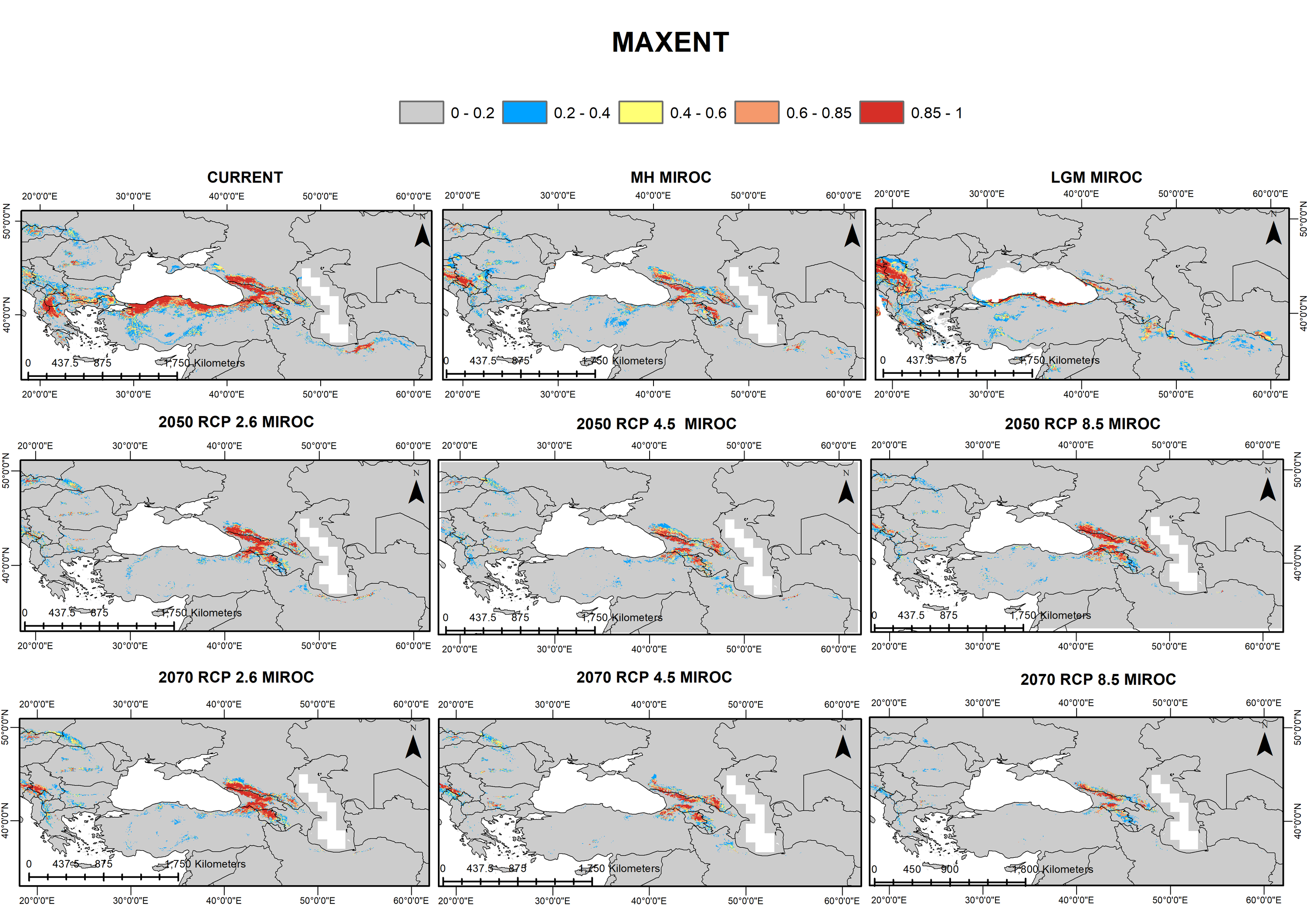

Supplement: S1 Fig — (TIF) [file pone.0242280.s003.tif]

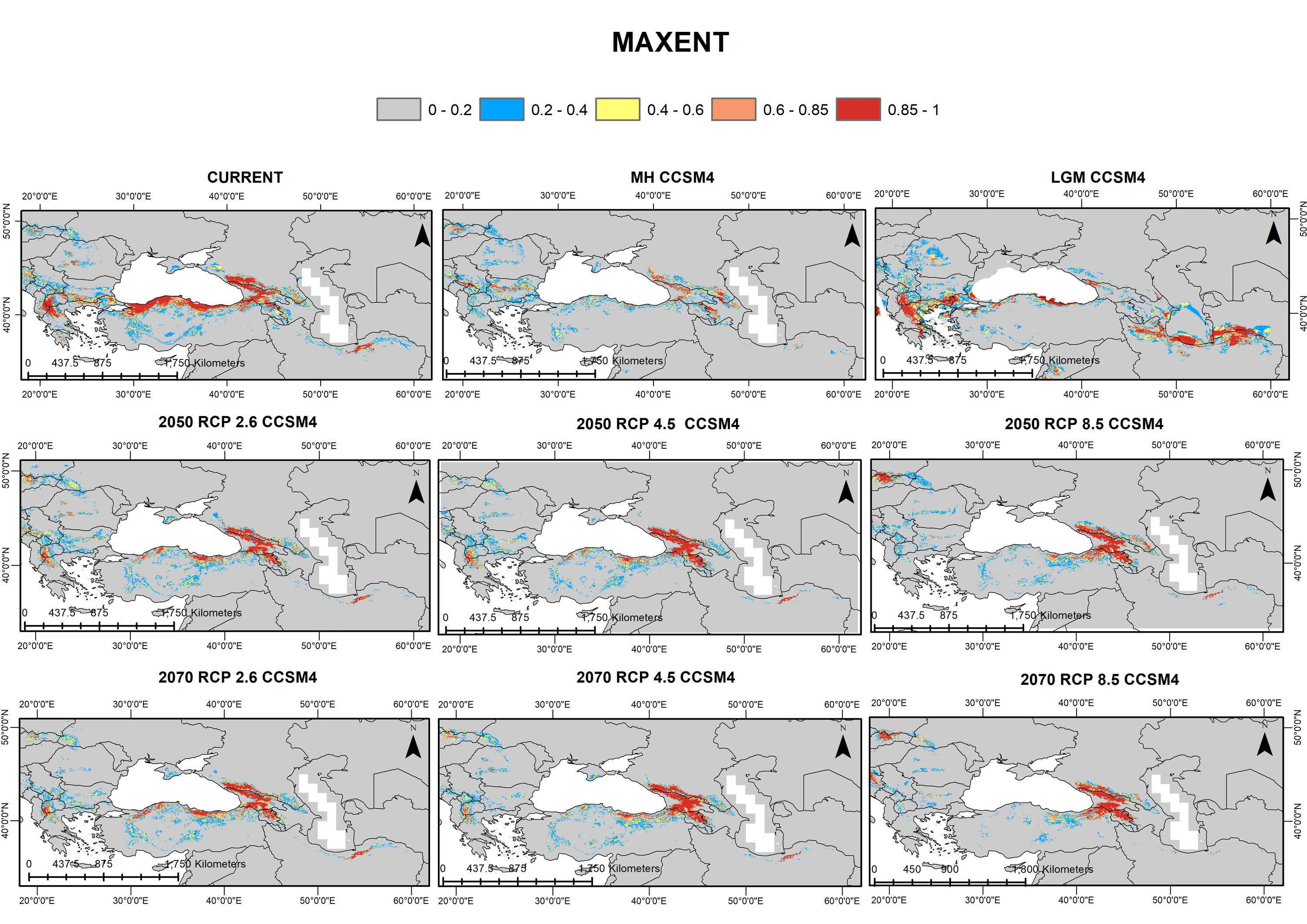

Supplement: S2 Fig — (TIF) [file pone.0242280.s004.tif]

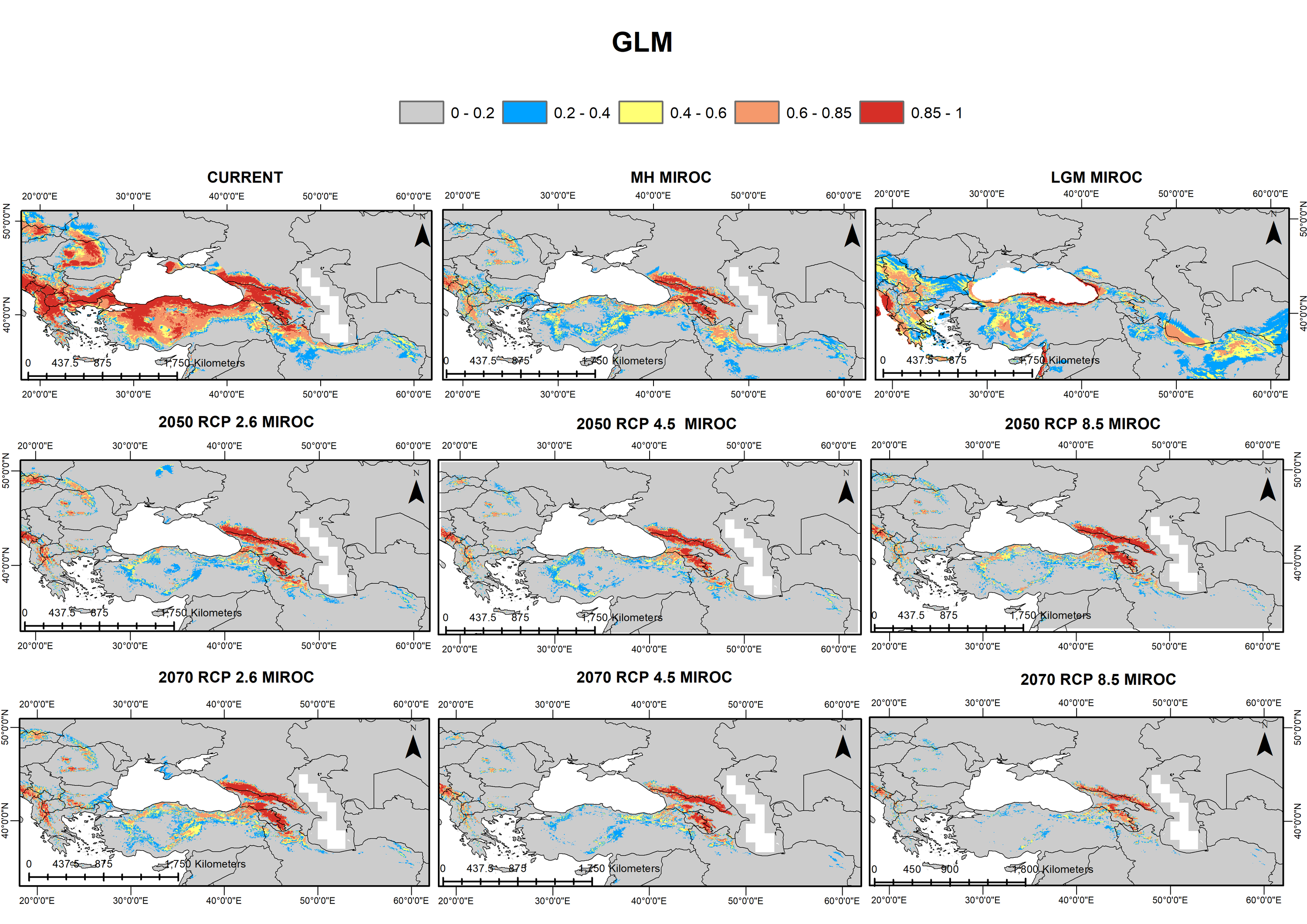

Supplement: S3 Fig — (TIF) [file pone.0242280.s005.tif]

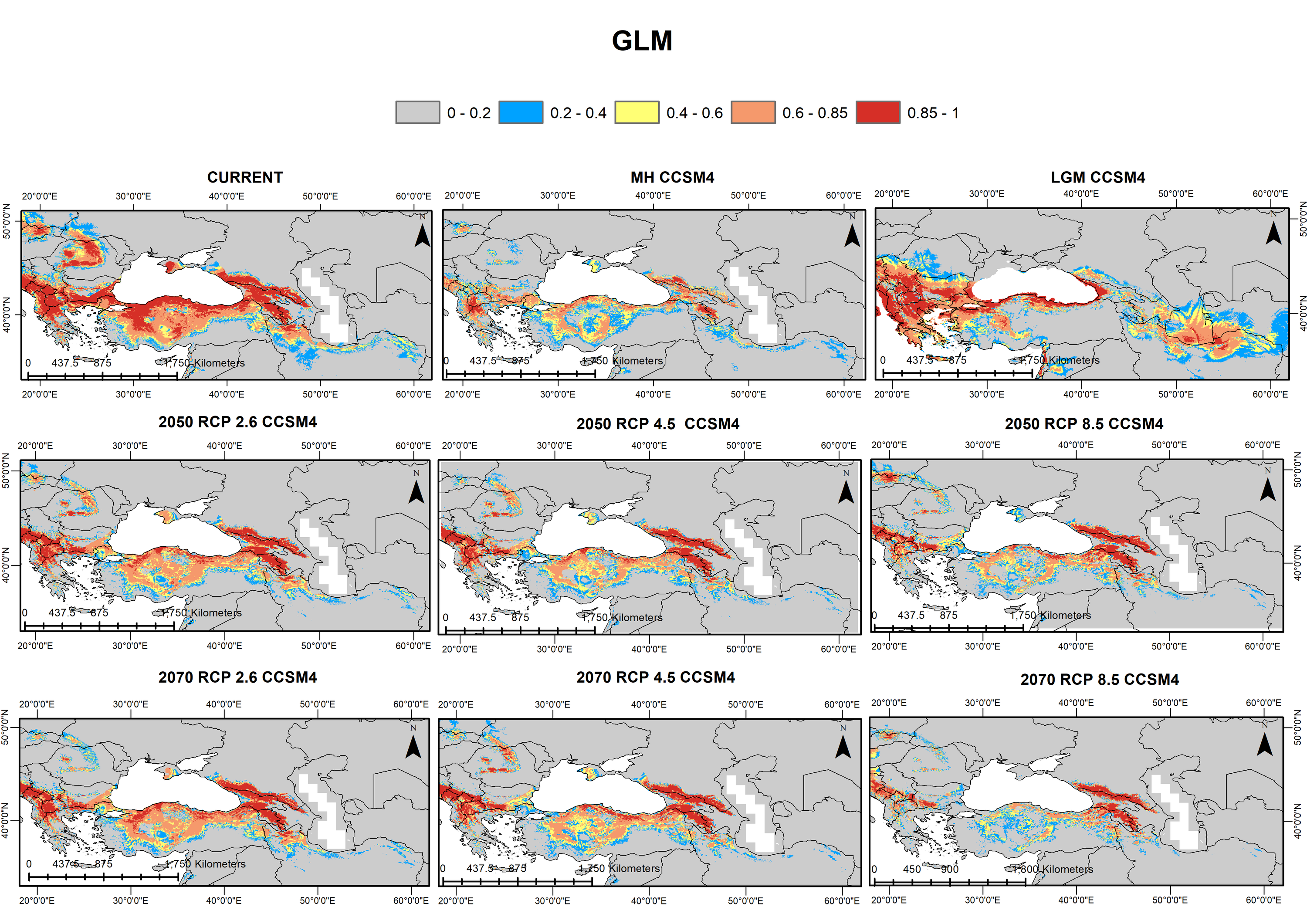

Supplement: S4 Fig — (TIF) [file pone.0242280.s006.tif]

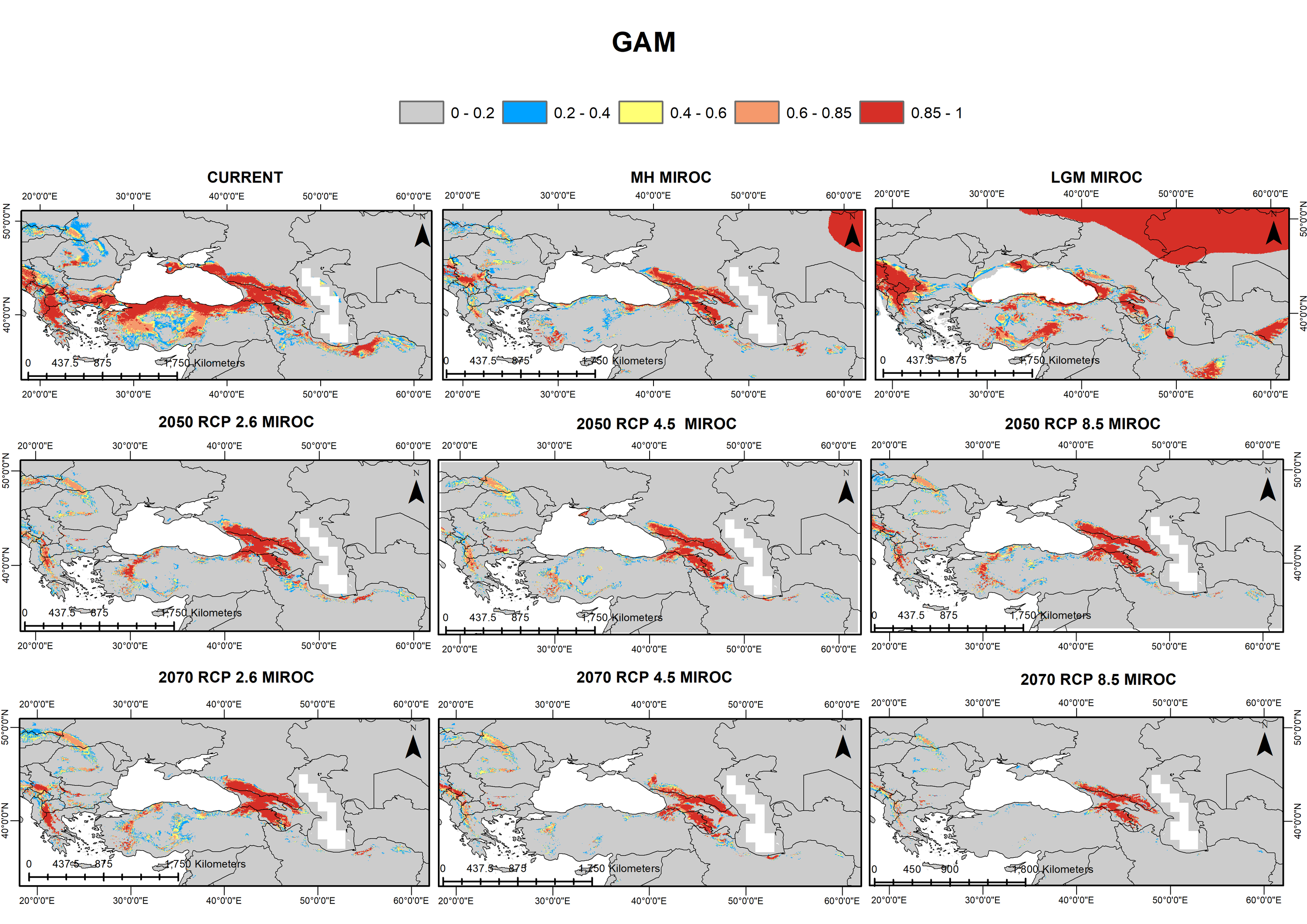

Supplement: S5 Fig — (TIF) [file pone.0242280.s007.tif]

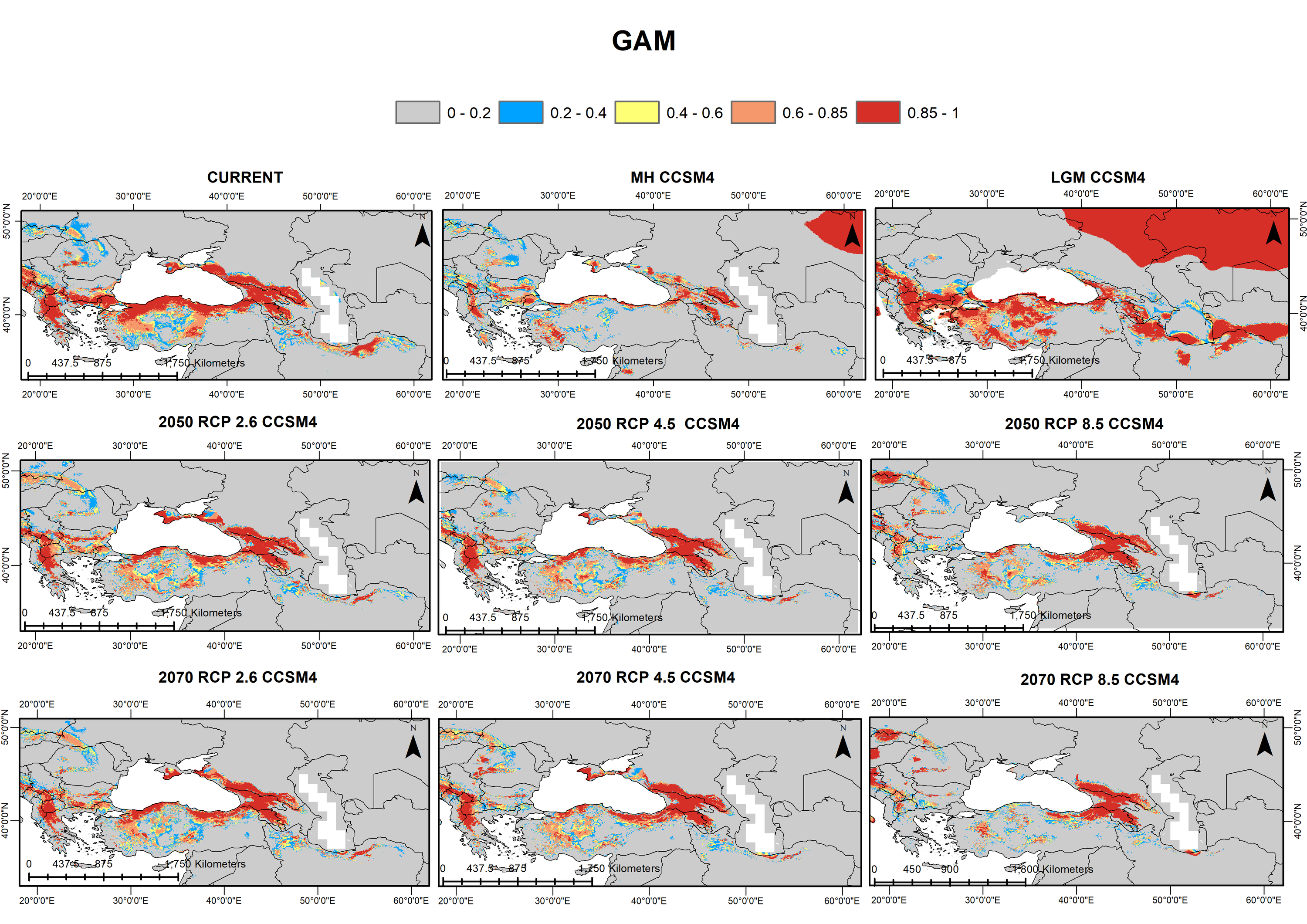

Supplement: S6 Fig — (TIF) [file pone.0242280.s008.tif]

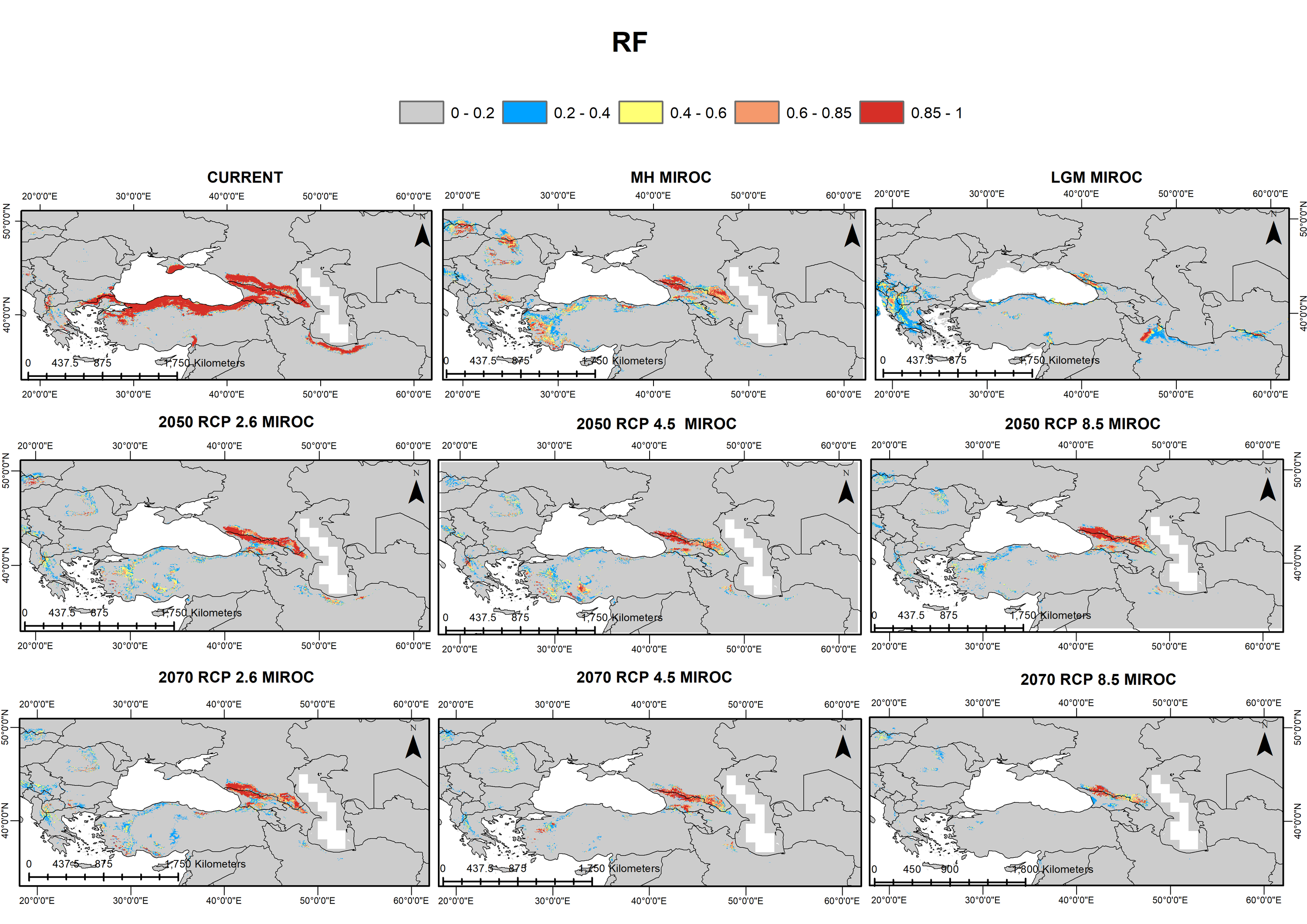

Supplement: S7 Fig — (TIF) [file pone.0242280.s009.tif]

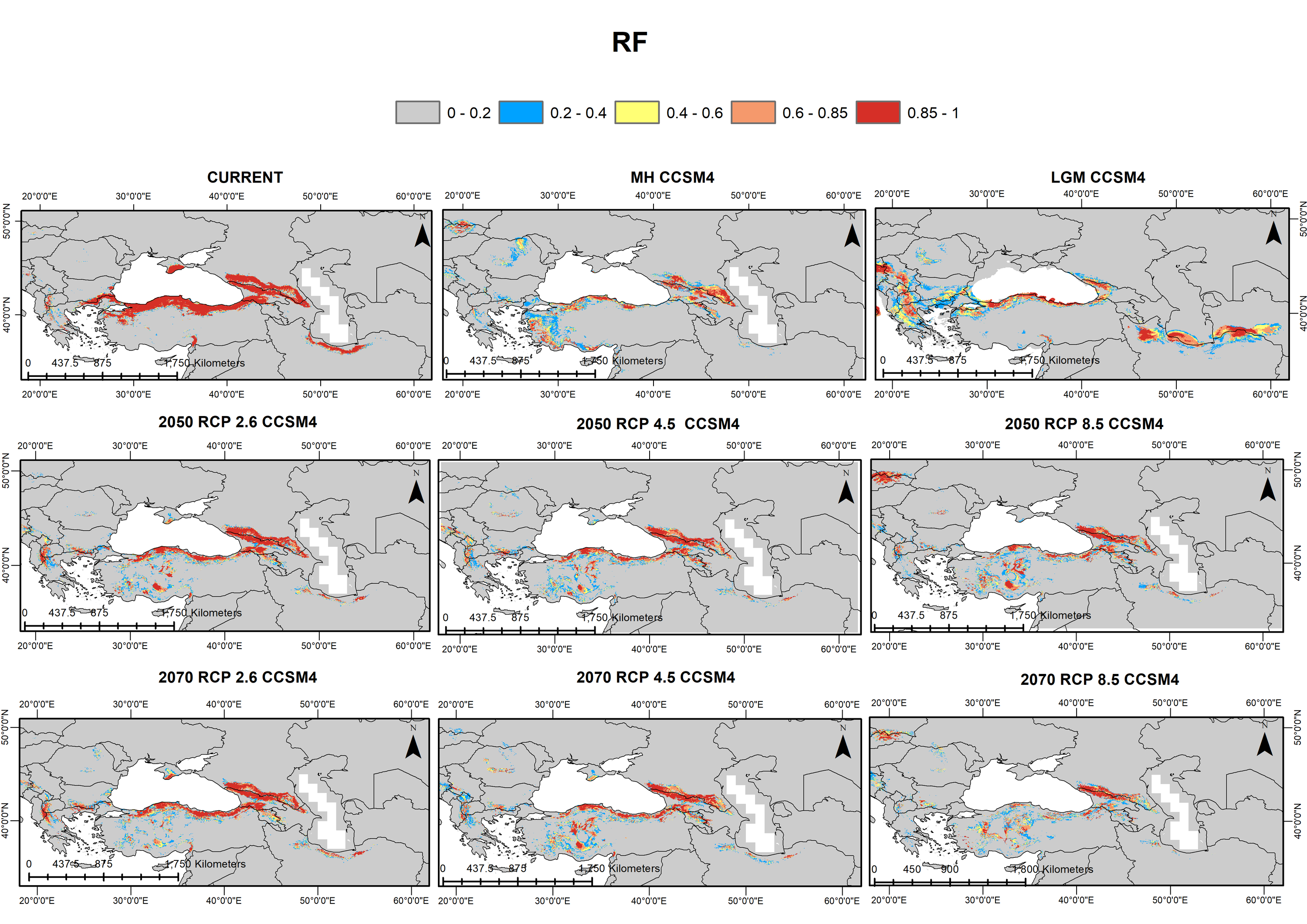

Supplement: S8 Fig — (TIF) [file pone.0242280.s010.tif]

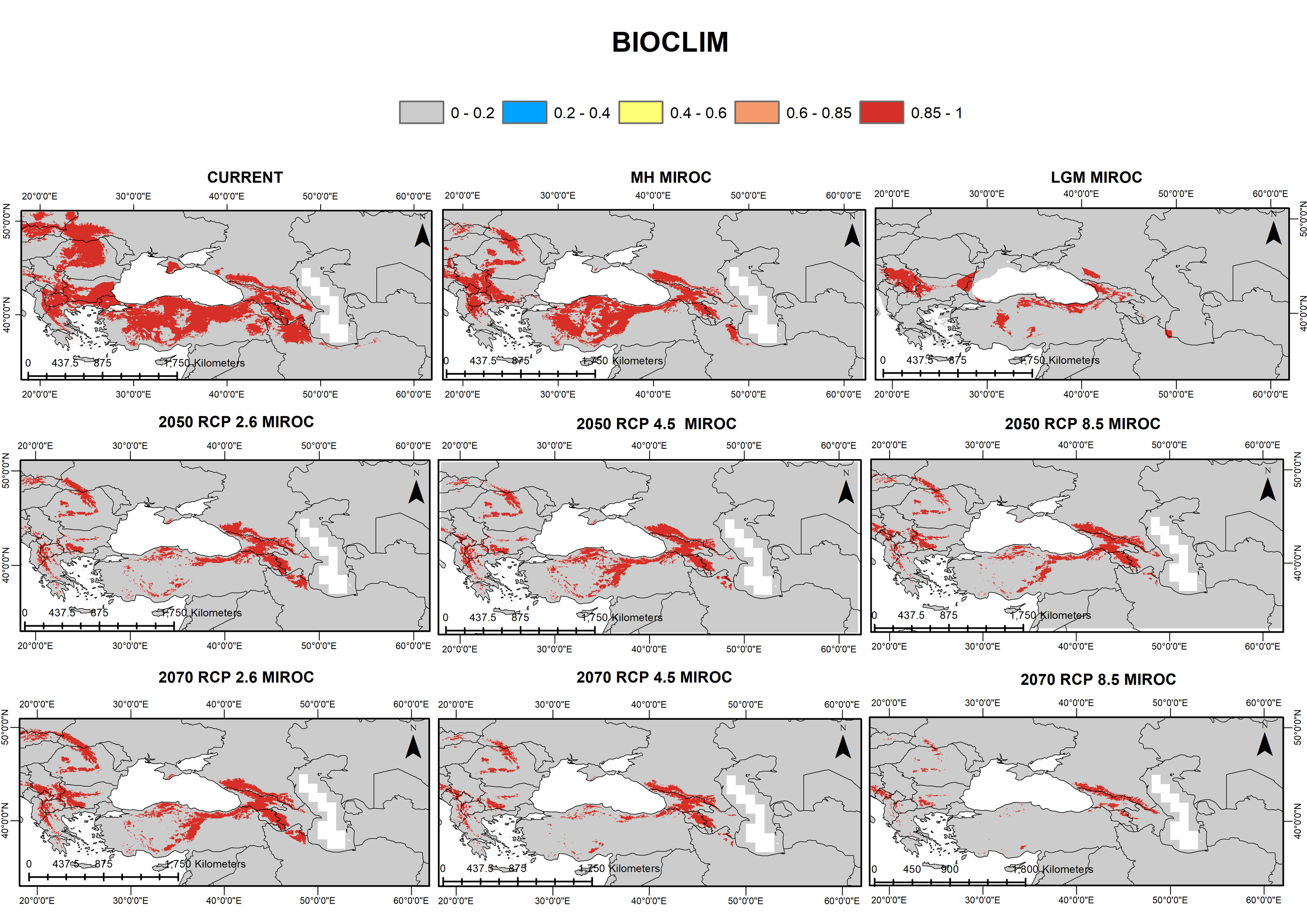

Supplement: S9 Fig — (TIF) [file pone.0242280.s011.tif]

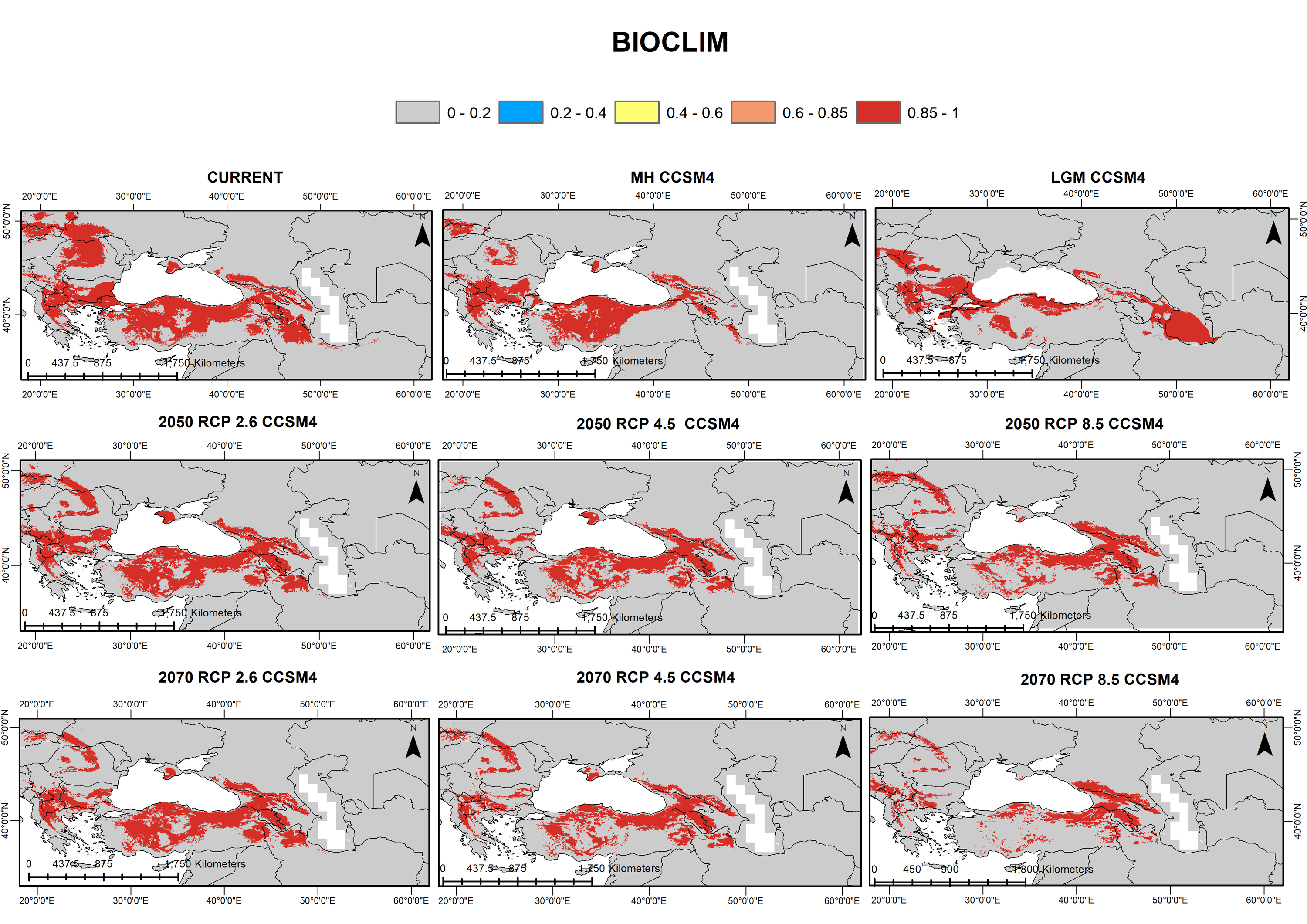

Supplement: S10 Fig — (TIF) [file pone.0242280.s012.tif]
